# Endotoxin induces proliferation of NSCLC in vitro and in vivo: role of COX-2 and EGFR activation

**DOI:** 10.1007/s00262-012-1341-2

**Published:** 2012-08-26

**Authors:** Katja Hattar, Rajkumar Savai, Florentine S. B. Subtil, Jochen Wilhelm, Anja Schmall, Dagmar S. Lang, Torsten Goldmann, Bastian Eul, Gabriele Dahlem, Ludger Fink, Ralph-Theo Schermuly, Gamal-Andre Banat, Ulf Sibelius, Friedrich Grimminger, Ekkehard Vollmer, Werner Seeger, Ulrich Grandel

**Affiliations:** 1 Department of Internal Medicine IV/V, University of Giessen and Marburg Lung Center (UGMLC), Klinikstrasse 33, Giessen, Germany; 2 Department of Radiotherapy and Radiooncology, Philipps-University, Marburg, Germany; 3 Clinical and Experimental Pathology, Research Center Borstel, Borstel, Germany; 4 Department of Pathology, University of Giessen and Marburg Lung Center (UGMLC), Giessen, Germany; 5 Department of Internal Medicine II, University of Giessen and Marburg Lung Center (UGMLC), Giessen, Germany; 6 Department of Internal Medicine II, Biostatistics Group, University of Giessen and Marburg Lung Center (UGMLC), Giessen, Germany; 7 Max-Planck Institute for Heart and Lung Research, Bad Nauheim, Germany

**Keywords:** Lung cancer, Infection, Endotoxin, Tumor proliferation, Inflammation

## Abstract

Lung cancer is frequently complicated by pulmonary infections which may impair prognosis of this disease. Therefore, we investigated the effect of bacterial lipopolysaccharides (LPS) on tumor proliferation in vitro in the non-small cell lung cancer (NSCLC) cell line A549, ex vivo in a tissue culture model using human NSCLC specimens and in vivo in the A549 adenocarcinoma mouse model. LPS induced a time- and dose-dependent increase in proliferation of A549 cells as quantified by MTS activity and cell counting. In parallel, an increased expression of the proliferation marker Ki-67 and cyclooxygenase (COX)-2 was detected both in A549 cells and in ex vivo human NSCLC tissue. Large amounts of COX-2-derived prostaglandin (PG)E_2_ were secreted from LPS-stimulated A549 cells. Pharmacological interventions revealed that the proliferative effect of LPS was dependent on CD14 and Toll-like receptor (TLR)4. Moreover, blocking of the epidermal growth factor receptor (EGFR) also decreased LPS-induced proliferation of A549 cells. Inhibition of COX-2 activity in A549 cells severely attenuated both PGE_2_ release and proliferation in response to LPS. Synthesis of PGE_2_ was also reduced by inhibiting CD14, TLR4 and EGFR in A549 cells. The proliferative effect of LPS on A549 cells could be reproduced in the A549 adenocarcinoma mouse model with enhancement of tumor growth and Ki-67 expression in implanted tumors. In summary, LPS induces proliferation of NSCLC cells in vitro, ex vivo in human NSCLC specimen and in vivo in a mouse model of NSCLC. Pulmonary infection may thus directly induce tumor progression in NSCLC.

## Introduction

Infections are considered to promote growth of human cancer. Roughly estimated, about 15 % of all malignancies worldwide can be attributed to infectious agents [1]. Although the contribution of different viruses to the development of malignancies has been well recognized, some chronic bacterial infections are also associated with tumor formation [2]. In this context, the most prominent example is *Helicobacter pylori*, which is an important risk factor for gastric cancer [3, 4].

In lung cancer, one of the leading causes of cancer-related death in the western hemisphere, the association between bacterial infections and cancer development is less obvious. Lung cancer patients frequently suffer from pulmonary infections, and the most common pathogens found in patients with lung cancer are gram-negative bacteria such as *Haemophilus influenzae* and *E. coli* [5, 6]. Although pulmonary infections have been related to a reduction in the median survival of patients with lung cancer [7], it is not clear whether bacterial infections worsen prognosis of lung cancer by actually accelerating tumor growth and metastasis formation. However, it is well established that persistent inflammation can activate cancer growth [8, 9], and in NSCLC, a prominent role for COX-2-derived lipid mediators has been postulated in this context [10, 11]. In vivo, COX-2 protein and mRNA levels are elevated and are associated with a poor prognosis in lung adenocarcinoma [12, 13]. In vitro, overexpression of COX-2 directly increases survival of lung adenocarcinoma cell lines [14]. PGE_2_ is the major COX-2-derived metabolite up-regulated in human lung cancer tissue and cell lines [15, 16]. Direct inhibition of apoptosis and an EGFR-associated signaling have been characterized as molecular mechanisms of PGE_2_-induced tumor growth [17].

Regarding NSCLC, COX-2 expression and PGE_2_ production in epithelial cancer cell lines have been shown to be induced by benzo[a]pyrene, a potent carcinogen contained in cigarette smoke [18]. In bronchial epithelial cells, however, COX-2 is also induced by endogenous and exogenous proinflammatory stimuli such as the bacterial membrane glycolipid LPS [19, 20], suggesting a relevant role for infectious agents in this context. In general, cellular activation by LPS is initiated via the CD14 surface receptor, a GPI-anchored glycoprotein [21] and TLRs, such as TLR4 [22, 23]. However, some LPS types, mainly from non-enterobacteria are recognized by TLR2, presumably due to differences in the lipid A component [24].

In gastric cancer, the expression of different TLRs enables gastric carcinoma cells to interact with *Helicobacter pylori* [25]. This interaction may be followed by the production of tumor-promoting factors such as IL-8. Most importantly, an up-regulation of TLR4 expression was recently demonstrated in human adenocarcinoma of the lung in vivo, and TLR4 expression levels correlated with malignancy [26]. Thus, specific interactions between bacterial pathogens such as LPS and tumor cells may actually occur in NSCLC. However, the consequences of such interactions for tumor cell biology are less clear.

In the current study, we focused on the effects of bacterial endotoxin in vitro on proliferation of A549 cells, a cell line derived from human lung adenocarcinoma, in an ex vivo short-term cultivation model designated short-term stimulation of tissues (STST) using human specimens obtained from patients with NSCLC and in vivo in the subcutaneous A549 adenocarcinoma mouse model. In essence, we found that LPS strongly induces proliferation in these experimental models, which was mediated by COX-2 activation. Furthermore, interference with CD14, TLR4 and EGFR attenuated the proliferative response to LPS. Thus, our data suggest that LPS exposure as a consequence of pulmonary infections could potentially accelerate tumor progression in lung cancer.

## Materials and methods

### Cell culture

The A549 human lung adenocarcinoma cell line was obtained from the American Type Culture Collection (ATCC, Rockville, MD, USA) and cultured at 37 °C in a humidified atmosphere (95 % air, 5 % CO2). All cell culture media and supplements were purchased from Gibco (Eggenstein, Germany) unless otherwise indicated. The cells were kept in Dulbecco’s modified Eagle’s medium (DMEM/F12), supplemented with 10 % FCS, 2 mM l-glutamine, 10^5 ^U/l penicillin and 100 mg/l streptomycin. Cells were grown to confluence and subcultured every 2–3 days and split at a ratio of 1:10.

### Ex vivo cultivation and stimulation of human lung cancer tissues

Three specimens of human NSCLC of adenocarcinoma type were cultured using a novel short-term tissue cultivation model ex vivo as previously reported [27]. Briefly, vital tissue samples were cultured in 2 ml RPMI 1640 supplemented with 10 % FCS at 37 °C and 5 % CO2 for 16 h in the presence or absence of 10 μg/ml of a highly purified LPS from *E. coli* F515 (kindly provided by Prof. Otto Holst, Immunochemistry Group, Research Center Borstel, Germany) [28]. After termination of the cultivation period [27], the specimens were fixed by the HOPE-technique and embedded in paraffin as described elsewhere [29]. Accordingly, the tissue samples were always deparaffinized for subsequent analyses (immunohistochemistry and molecular analysis).

### A549 adenocarcinoma mouse model

Tumor growth was assessed by subcutaneous injection of A549 cells (2.5 × 10^6^ cells/200 μl in PBS) into 8-week-old female BALBc/c nu/nu mice. These mice were purchased from Charles River (Sulzfeld, Germany) and kept under specific pathogen-free conditions. Immediately before subcutaneous injection, A549 cells were treated with highly purified *E.coli* LPS F515 (*n* = 6) or sham incubation with PBS (control, *n* = 8) was performed. Animals were handled in accordance with the European Community recommendations for experimentation. The size of the tumor was measured at days 4, 8 and 12 after implantation by Mitutoyo digital calipers (Mitutoyo Ltd., UK), as previously described [30]. The tumor volume (TV) was calculated by the formula TV (mm^3^) = (*L* × *W* ^2^)/2, where *L* is the longest dimension of the tumor (in mm) and *W* is the shortest dimension of the tumor (in mm). Each tumor measurement was taken in triplicate by two different investigators (K.H. and R.S.)

### MTS assay

The MTS assay (CellTiter 96@ Aqueous One Solution Cell Proliferation Assay, Promega, Mannheim, Germany) quantifies the metabolic activity of cells. This assay is based upon the cleavage of the yellow 3-(4, 5-dimethylthiazol-2-yl)-5-(3-carboxymethoxyphenyl)-2-(4-sulfophenyl)-2H-tetrazolium, inner salt (MTS) to purple formazan by metabolic active cells. The production of the colored formazan product is directly proportional to the number of viable cells in culture [31]. Based on these data, the MTS assay is widely used for the assessment of cellular proliferation.

**Fig. 1:**
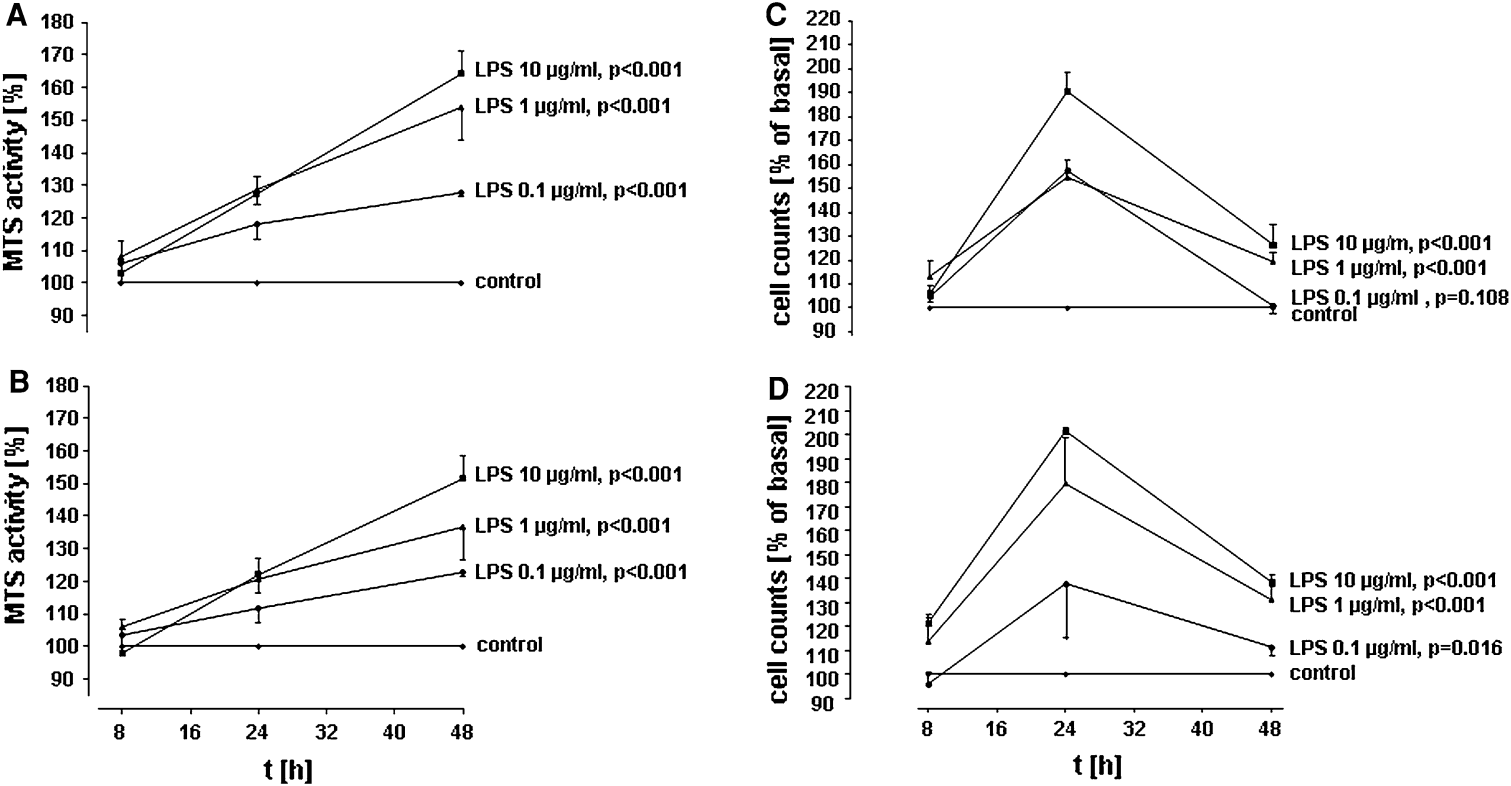
Time-and dose-dependent induction of A59 proliferation by LPS. A549 cells were incubated with various concentrations of LPS from *E. coli* 0111:B4 (**a**/**c**) or highly purified LPS from *E. coli* F515 (**b**/**d**) or sham incubation was performed (*control*). A549 proliferation was assessed by measuring MTS activity (**a**/**b**) and automatic cell counting (**c**/**d**). All data are expressed as percentage of unstimulated cells (*control*). Mean ± SEM of at least four independent experiments are given

**Fig. 2:**
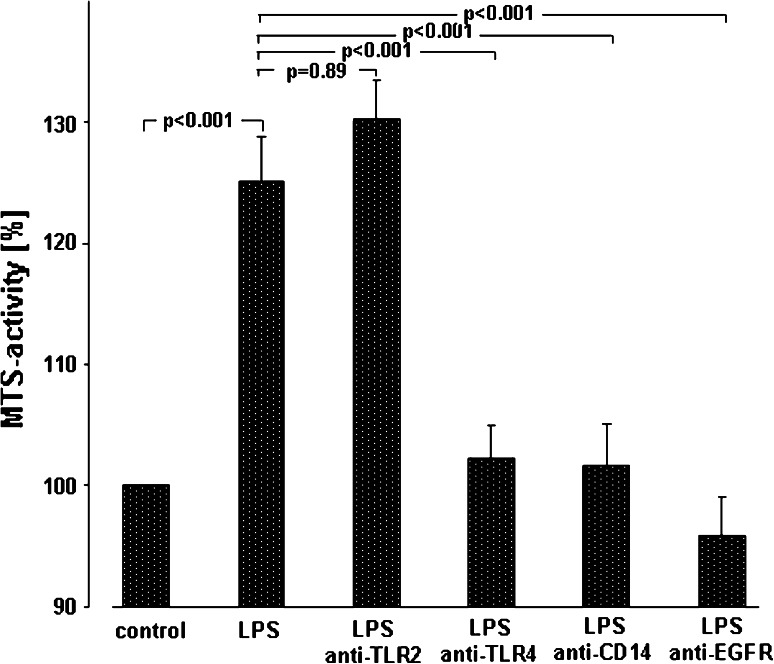
Mechanisms of LPS-induced A549 proliferation. A549 cells were either sham-incubated (*control*) or exposed to 10 μg/ml of LPS (*n* = 3 for LPS 0111:B4 and *n* = 3 for LPS F515, total *n* = 6) in the absence or presence of neutralizing antibodies targeting TLR2, TLR4, CD14 or EGFR. After 24 h of incubation proliferation was quantified by determining MTS activity. All data are expressed as percentage of unstimulated cells (*control*). Mean ± SEM of six independent experiments are given

In brief, A549 cells were seeded on 96-well plates (2,500 cells/well) and maintained in culture for 24 h before LPS stimulation. Then, medium was exchanged, and cells were kept in RPMI containing 1 % FCS at a total volume of 200 μl/well. A549 cells were stimulated with different concentrations of LPS (*E. coli* LPS 0111:B4, Sigma, Deisenhofen, Germany or highly purified *E.coli* LPS F515) for various time periods or sham incubation (control) was performed. For Fig. 1a with LPS from *E. coli* 0111:B4, at least five independent experiments were performed, and for Fig. 1b with LPS from *E. coli* F515, at least four independent experiments were performed. In an additional series of experiments in Fig. 2, function-blocking antibodies targeting TLR2 (clone TL2.1, e-Bioscience, San Diego, CA, USA), TLR4 (clone HTA 125, e-Bioscience, San Diego, CA, USA), CD14 (MY-4, Coulter Immunotech, Hamburg, Germany), EGFR (Cetuximab, Merck Serono, Germany) or COX inhibitors (indomethacin, Sigma, Deisenhofen, Germany and NS-398, Calbiochem, La Jolla, CA, USA) were applied simultaneously to LPS. For these inhibitor studies, at least six independent experiments were performed (at least 3 both for LPS 0111:B4 and for LPS F515, respectively).

At the end of the incubation, 20 μl of MTS solution were added to each well and plates were again incubated for 2.5 h at 37 °C. Absorbance was read at 490 nm, background readings were subtracted from the sample wells, and data were expressed as percentage of controls (sham-incubated cells). All samples were run in triplicate, and all measurements were taken twice after 2.5 h of incubation with the MTS reagent. All data were expressed as percentage increase in MTS activity compared to unstimulated cells (controls), which were set to 100 %.

### Assessment of cellular proliferation by cell counting

For cell counting, A549 cells were seeded on 24-well plates (50,000 cells/well) and maintained in culture for 24 h before LPS stimulation. Then, medium was exchanged, and cells were kept in RPMI containing 1 % FCS at a total volume of 500 μl/well. A549 cells were stimulated with different concentrations of LPS (*E. coli* LPS 0111:B4 or *E. coli* LPS F515) or sham-incubated (control). For each LPS type in Fig. 1c, d, at least four independent experiments were performed. At the end of the incubation period, the medium was removed, and cells were washed, detached by treatment with 0.5 % trypsin–EDTA, resuspended in a stop-solution containing 20 % FCS in PBS and finally counted by the cell counter-analyzer system Casy Model TT (Innovatis AG, Reutlingen, Germany). Data were expressed as percentage of controls (sham-incubated cells), which were set to 100 %. All samples were performed in triplicate, and all measurements were taken three times in 200 μl cell casyton suspension each. The orifice tube had an aperture size of 150 μm.

### Measurement of PGE_2_ and thromboxane A_2_

**Fig. 3:**
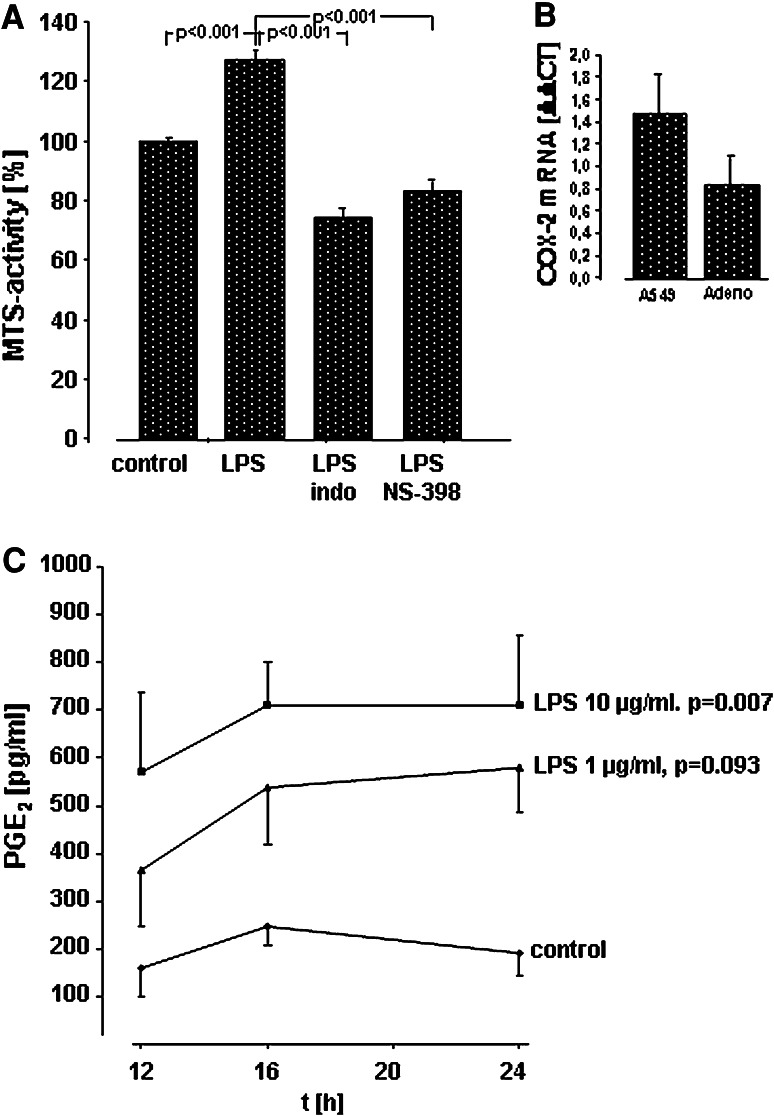
Activation of COX-2 and release of PGE_2_ in A549 cells and human lung cancer tissue in response to LPS. **a** Effect of COX inhibitors on LPS-induced proliferation of A549 cells in vitro. A549 cells were either sham-incubated (*control*) or exposed to 10 μg/ml of LPS (*n* = 3 for LPS 0111:B4 and *n* = 3 for LPS F515, total *n* = 6) in the absence or presence of indomethacin (*indo*) or the COX-2 inhibitor NS-398. After 24 h of incubation proliferation was quantified by determining MTS activity. All data are expressed as percentage of unstimulated cells (*control*). Mean ± SEM of six independent experiments are given. **b** Expression of COX-2 mRNA in response to LPS in A549 cells and human lung cancer tissue. A549 cells and specimen of human adenocarcinoma were either sham-incubated or exposed to 10 μg/ml LPS F515. After 16 h, mRNA was extracted and subjected to quantitative reverse transcriptase polymerase chain reaction. The ΔΔCT values represent relative expression of COX-2 mRNA normalized to the internal reference HPRT mRNA in LPS versus unstimulated A549 cells or lung adenocarcinoma (*Adeno*) tissue. Mean values ± SEM, originating from four independent experiments, each performed in duplicate are given. **c** Release of PGE_2_ in A549 cells in response to LPS. A549 cells were either sham-incubated (*control*) or exposed to the given concentrations of LPS (at least *n* = 3 for LPS 0111:B4 and *n* = 3 for LPS F515, total at least *n* = 6) for various time periods. 8 h before the end of the incubation period, and AA was added. PGE_2_ release into the cell supernatant is given in pg/ml. Data are expressed as mean ± SEM of at least six independent experiments

PGE_2_ and thromboxane (Tx)B_2_, the stable hydrolysis product of TxA_2_, were quantified in a commercial ELISA system (R&D Systems, Wiesbaden, Germany) according to the manufacturer’s instructions. For these experiments, A549 cells (50,000 cells/well) were seeded on 24-well plates and grown to confluence. Confluent monolayers were washed twice and kept in RPMI containing 1 % FCS at a total volume of 500 μl/well. Then, incubation with different concentrations of LPS (from *E. coli* 0111:B4 or *E. coli* F515) in the absence or presence of the respective antibodies or COX inhibitors for various time periods, or sham incubation (control) was performed. All samples were performed as duplicate. For the time–response curve in Fig. 3c, as well as for the inhibitor studies in Fig. 3a, at least six independent experiments were performed (at least 3 both for LPS 0111:B4 and for LPS F515, respectively). At the end of the incubation period, medium was exchanged, and cells were washed twice and kept in RPMI containing 1 % FCS and further incubated for 8 h with 5 μM arachidonic acid (AA, Sigma, Deisenhofen, Germany). Then, cell supernatants were harvested, cell debris was removed by centrifugation at 13.000×*g*, and samples were stored at −20 °C until further processing. The measurement of PGE_2_ and TxB_2_ release was taken by ELISA technique, according to the manufacturer’s protocols and is expressed in pg/ml. All samples were performed as duplicate, and each sample was measured twice.

### RNA isolation and real-time RT-PCR

For quantification of COX-2 in fig. 3b, Ki-67 and PCNA mRNA, experiments with A549 cells (50,000 cells/well) or short-term stimulation of lung cancer tissues were performed as described above for 16 h. Total RNA was extracted from cells and lung cancer tissues with TRIzol reagent (Invitrogen, Karlsruhe, Germany) or RNeasy minikit (Qiagen, Hilden, Germany), following the manufacturer’s protocols. The yield of extracted RNA was determined by Nano Drop (PeqLab, Erlangen, Germany). After digesting residual DNA with DNase (Invitrogen, Karlsruhe, Germany), cDNA was synthesized by RT (Promega, Mannheim, Germany or Applied Biosystems, Darmstadt, Germany). Real-time PCR was performed using 1 μg of cDNA, SYBR Green PCR Master Mix (Invitrogen, Karlsruhe, Germany) and 0.05 M forward/reverse primers; specific primers used for sequence detection were as follows:

- for HPRT
- 5′GGTCCTTTTCACCAGCAAGCT3′8 (forward) and 5′TGACACTGGCAAAACAATGCA3′ (reverse),
- for PCNA
- 5′TTTTCTGTCACCAAATTTGTACCTC3′ (forward) and 5′CTGCATTTAGAGTCAAGACCCTTT3′ (reverse)
- for Ki-67
- 5′AGAAGACAGTACCGCAGATGA3′ (forward) and 5′CGGCTCACTAATTTAACGCTGG3′ (reverse),
- for COX-2
- 5′ATCATAAGCGAGGGCCAGCT3′ (forward 101 bp) and 5′AAGGCGCAGTTTACGCTGTC3′ (reverse 101 bp).

Real-time reactions were carried out on the Stratagene Mix Pro 3000 P (Agilent Technologies Inc) or the Sequence Detection System 7900 HT (Applied Biosystems) with following cycle conditions: denaturation, 95 °C for 10 min, 40 cycles with denaturation at 95 °C for 30 s, annealing at 58–60 °C for 30 s and extension at 72 °C for 30 s. To ensure single-product amplification, a dissociation curve was generated for each gene and the threshold cycle (Ct values) for each gene was determined. The comparative 2−ΔΔCt method was used to analyze mRNA-fold changes between control and LPS, which was calculated as ratio = 2−(ΔCt control−ΔCt LPS). Ct is the cycle threshold and ΔCt (Ct target − Ct reference) is the CT value normalized to the housekeeper gene HPRT obtained for the same cDNA samples [32, 33]. All samples were performed in triplicate, and all measurements were taken three times.

### Histological examination of explanted tumors from the A549 adenocarcinoma mouse model

The tumors were frozen in liquid nitrogen and stored at −80 °C. For immunofluorescence, six LPS-treated tumors and eight unstimulated tumors were analyzed. 5 μm whole tumor cross-sections were cut from the central part. Immunofluorescence staining has previously described in detail [34]. Briefly, whole tumor cross-sections were fixed with methanol and acetone (1:1) for 5 min and washed three times with PBS containing 0.1 % BSA and 0.2 % Triton X-100. The unspecific binding sites were blocked with 3 % BSA in PBS for 1 h. For Ki-67, slides were stained with a polyclonal rabbit anti-human nuclear Ki-67 (Abcam Ltd.332, ab833, Cambridge, UK, dilution 1:100 [35]. The secondary antibody, consisting of a goat anti-rabbit IgG (Alexa Fluor 488, Molecular Probes, Eugene, Oregon, USA) was applied at 1:1000. Each section was counterstained for 5 min with 40, 6-diamidino-2-phenylindole (DAPI, Sigma, Deisenhofen, Germany) and mounted with fluorescent mounting medium (Dako, Hamburg, Germany). The cyrosections were also stained with hematoxylin–eosin (H&E) [36].

Microscopic analyses were performed using a fluorescence microscope (Leica DMLA Q550/W, Leica Microsystems, Bensheim, Germany) and Leica Q-Win standard software for quantification. The whole tumor cross-sections were sequentially scanned. After the scanning procedure, the Ki-67-positive signal of the whole tumor cross-section was detected, and this positive area was measured and related to the whole DAPI-positive area of the same tumor cross-section [37].

### Expression of Ki-67 in human lung cancer tissue

Ki-67 was analyzed by immunohistochemistry (IHC) as described earlier [38]. Primary antibody MIB-1 (Dako, Glostrup, Denmark, 333 ng/ml) was used in a final dilution of 1:100. After 30 min at room temperature, visualization was performed by horseradish-peroxidase labeled streptavidin–biotin technique (LSAB2™, Dako, Denmark) diluted 1:3 and using 3-Amino9-Ethylcarbazole/H_2_O_2_ as chromogen. Slides were counterstained with Mayer’s hemalum and mounted with Kayser’s glycerine gelatine. Negative controls were run by omitting the primary antibody.

### Statistics

**Fig. 4:**
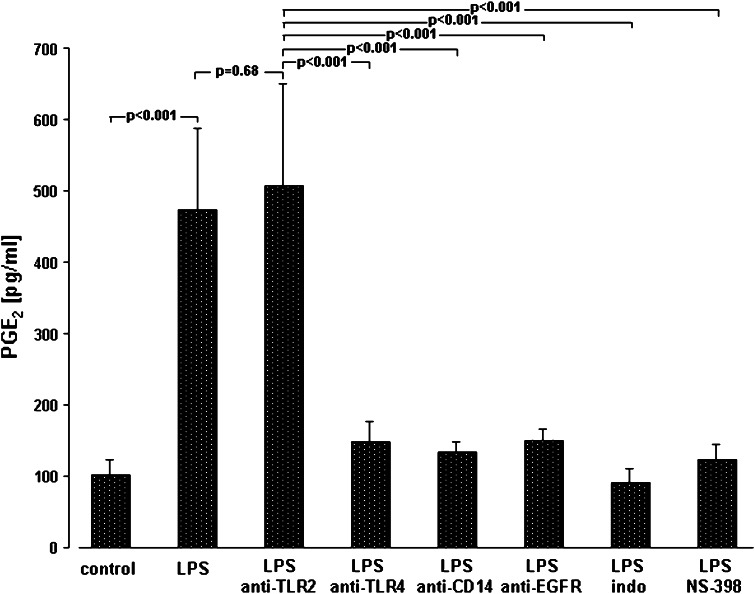
Mechanisms of LPS-induced PGE_2_ synthesis. A549 cells were either sham-incubated (*control*) or exposed to 10 μg/ml of LPS (*n* = 3 for LPS 0111:B4 and *n* = 3 for LPS F515, total *n* = 6) in the absence or presence of neutralizing antibodies targeting TLR2, TLR4, CD14 and EGFR, or the COX inhibitor indomethacin (*indo*) and the specific COX-2 inhibitor NS-398 for 24 h. 8 h before the end of the incubation period, and AA was added. PGE_2_ release into the cell supernatant is given in pg/ml. Data are expressed as mean ± SEM of at least six independent experiments

**Fig. 5:**
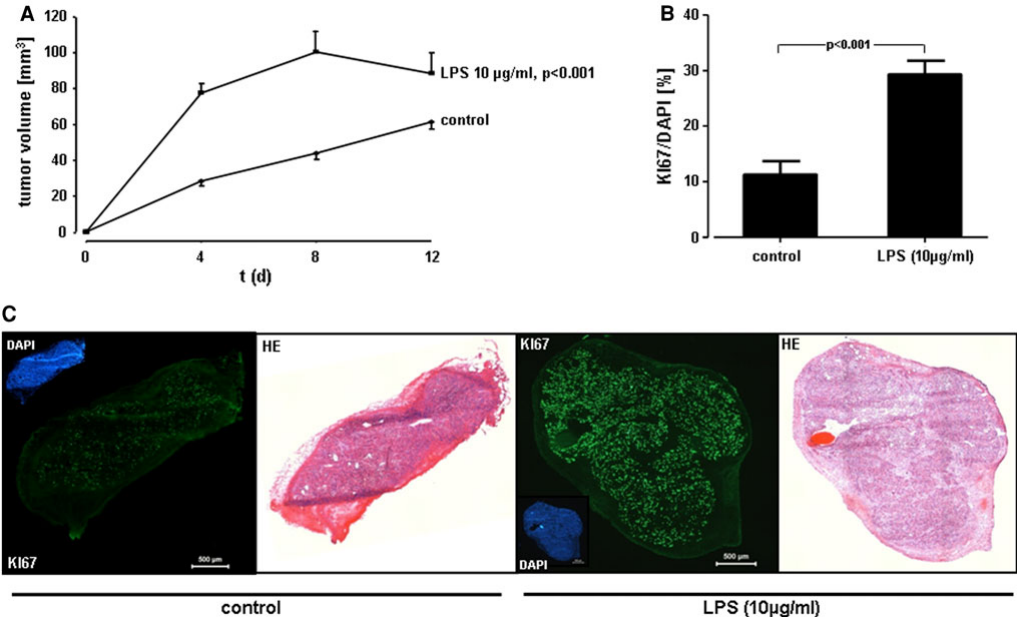
Proliferative response of A549 cells in vivo. **a** In vivo tumor growth. A549 cells were exposed to 10 μg/ml of LPS F515, or sham incubation was performed. Immediately after treatment, cells were injected subcutaneously into 8-week-old female BALBc nu/nu mice. At indicated time points, the size of the tumor was measured by Mitutoyo digital calipers and is given in mm^3^. Data are expressed as mean ± SEM (*n* = 8 for controls and *n* = 6 for LPS). **b** Immunohistofluorescent analysis of Ki-67 in cryosections from A549 tumors. Quantitative analysis of Ki-67 relative to DAPI (%) from the experiments described in (**a**). Data reflect the mean ± SEM (*n* = 8 for controls and *n* = 6 for LPS). **c** Immunofluorescent and H&E staining. Representative images of untreated (*control*) versus LPS-stimulated (10 μg/ml) A549 tumors and of the corresponding DAPI, Ki-67 and the H&E staining, respectively. The *scale bar* corresponds to 500 μm

Unlike otherwise indicated, data are given as the relative changes compared to control values and expressed as the mean ± SEM. Raw data were analyzed with R [39]. Linear mixed models were calculated using the package “lme” [40]. Raw data from time series were analyzed using the area-under-the-curve (AUC) approach. AUC values were calculated using the trapezoid rule. Percentages were analyzed using beta regression [41]. Linear mixed models were used for Figs. 1a, b, 2, 3a and 4. Linear models were used for Figs. 1c, d, 3c and 5a. Beta regression was used for Fig. 5b. Residuals of the models were checked for normal distribution, variance homogeneity and influential points. Reported p values are not corrected for multiple testing. Unless otherwise stated, p values below 0.05 keep a family-wise error rate of 5 % (i.e., they would be <0.05 after Bonferroni correction).

## Results

### Induction of a time- and dose-dependent proliferation of A549 cells by LPS

The A549 monolayers were incubated with different concentrations of LPS (0.1, 1 and 10 μg/ml) for various time periods (8, 24, 48 h). Two different endotoxins were used, either *E. coli* LPS 0111:B4 (Fig. 1a/c) or highly purified *E.coli* LPS F515 (Fig. 1b/d). Both LPS preparations stimulated the proliferation of A549 cells in a time- and dose-dependent manner, as quantified by MTS assay and cell counting when compared to unstimulated controls. The maximal increase in metabolic activity was induced by 10 μg/ml endotoxin stimulation. In response to LPS from *E. coli* 0111:B4, MTS activity was increased to 129 % after 24 h of stimulation and to 157 % after 48 h of incubation (Fig. 1a). Similiar results were obtained for highly purified LPS from *E. coli* F515 (Fig. 1b), with an increase in MTS activity to 122 % after 24 h and to 151 % after 48 h. Also, cell numbers were maximally increased upon exposure to 10 μg/ml endotoxin. After 24 h, LPS from *E. coli* 0111:B4 elicited an increase in cell numbers to 191 % and to 127 % after 48 h (Fig. 1c), while stimulation with LPS from *E.coli* F515 was equally effective: cell counts were elevated to 201 % after 24 h and to 138 % after 48 h, respectively. Continuous cellular growth was observed during the incubation period (untreated cells were counted as 745 cells after 8 h, 1,060 cells after 24 h and 3,978 cells after 48 h). Moreover, Ki-67 mRNA, as normalized to transcription of the internal standard gene HPRT was up-regulated 3.52 ± 0.52-fold, and PCNA mRNA was up-regulated 5.61 ± 0.88-fold in A549 cells after stimulation with 10 μg/ml LPS (*n* = 6, *n* = 3 for LPS 0111:B4 and *n* = 3 for LPS F515).

### Mechanisms of LPS-induced A549 proliferation in vitro

In order to determine the molecular steps in A549 activation by endotoxin, studies with blocking antibodies targeting CD14 (MY-4, 5 μg/ml), TLR2 (TL2.1 10 μg/ml) or TLR4 (HTA125, 10 μg/ml) were performed. As depicted in Fig. 2, LPS increased metabolic activity to 127 % of unstimulated controls in the absence of blocking antibodies. However, this LPS-induced increase in metabolic activity was abolished in the presence of anti-CD14 (102 %) or anti-TLR4 (100 %), whereas blocking of TLR2 activity was not effective (128 %). In addition, targeting EGFR with cetuximab (10 μg/ml) suppressed the LPS-induced proliferation of A549 cells completely (96 %).

### Activation of COX-2 and release of PGE_2_ in response to LPS

As COX-2-dependent prostanoids play a central role in NSCLC proliferation, the role of COX activation in response to LPS was investigated. In the presence of both the non-specific COX inhibitor indomethacin (100 μM) and the COX-2-specific inhibitor NS-398 (10 μM), the LPS-induced cellular proliferation (127 %) was reduced below control levels (reduction to 74 % for indomethacin and to 83 % for NS-398 compared with unstimulated controls, Fig. 3a).

In parallel, COX-2 mRNA in A549 cells and in ex vivo stimulated human NSCLC tissue specimens was up-regulated as reflected by the positive ΔΔ CT values for COX-2 mRNA in response to LPS (Fig. 3b). Moreover, a time- and dose-dependent accumulation of PGE_2_ was detected in the supernatant of LPS-stimulated A549 cells (Fig. 3c). Again, the higher (10 μg/ml) endotoxin concentration was most effective and elicited an almost fivefold increase in PGE_2_ after 24 h. TxB_2_ was not released (data not shown). PGE_2_ synthesis in response to LPS could be effectively prevented by indomethacin (100 μM) and the specific COX-2 inhibitor NS-398 (10 μM). Both inhibitors diminished LPS-induced PGE_2_ release to control levels (Fig. 4). Corresponding to the LPS-activated proliferation of A549 cells, PGE_2_ release depended on ligation of TLR4 and CD14. Moreover, targeting EGFR also inhibited PGE_2_ release in response to LPS (Fig. 4).

### Tumor cell proliferation in response to endotoxin in vivo

In order to investigate the effect of LPS on tumor growth in vivo, LPS-stimulated (10 μg/ml) or unstimulated A549 cells were injected subcutaneously into BALBc nu/nu mice. All mice developed tumors at the site of injection. The size of tumor xenografts was measured over a 12 days period. Already after 4 days, tumor growth was 2.8-fold enhanced upon LPS stimulation. In fact, tumor size was 27 mm^3^ in unstimulated tumors (control) and 78 mm^3^ in LPS-stimulated tumors (Fig. 5a). These growth effects of LPS were observed up to 12 days after tumor cell transplantation. The increase in tumor size was accompanied by a significant up-regulation of the proliferation marker Ki-67, as assessed by immunofluorescence staining. In LPS-stimulated tumors, 30 % of cells expressed Ki-67 expression, while in unstimulated controls only 12 % of tumor cells were Ki-67 positive (Fig. 5b).

### Ki-67 expression in human lung cancer tissue after LPS exposure

Short-term stimulation of NSCLC tissue specimens using the ex vivo STST model [27] with 10 μg/ml LPS for 16 h revealed a twofold up-regulation of the proliferation marker Ki-67 as assessed by immunohistochemistry. Mean values of Ki-67 nuclear staining were 7.5 ± 3.8 % positive cells in untreated NSCLC specimens versus 15.0 ± 5.77 % positive cells in LPS-treated tissue specimens (*n* = 3).

**Fig. 6:**
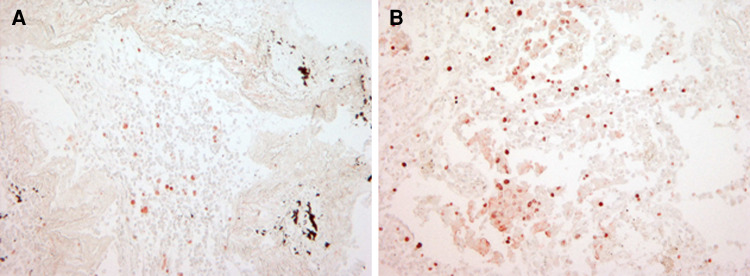
Expression of Ki-67 in human non-small cell lung cancer tissue in response to LPS. Human lung adenocarcinomas were cultivated in the absence or presence of 10 μg/ml LPS F515 (*n* = 3) for 16 h and subsequently treated with the novel HOPE-fixation technique and paraffin-embedding method. After deparaffinization, Ki-67 protein expression was assessed by IHC. One representative example of both an unstimulated (**a**) and LPS-stimulated (**b**) human NSCLC specimen of adenocarcinoma type is shown (magnification ×200)

Immunohistochemical staining of Ki-67 in a representative human NSCLC specimen of adenocarcinoma type in the absence or presence of LPS is depicted in Fig. 6.

## Discussion

Pulmonary infections are frequently encountered in lung cancer and may worsen prognosis in advanced stages of the disease. While therapy of lung cancer is often hampered by recurrent pulmonary infections, it is still unknown whether lung cancer growth and progression are actually accelerated by bacterial infections of the lung. In the present study, we demonstrated that purified endotoxin, the main pathogenicity factor of gram-negative bacteria, promotes tumor progression in A549 cells in vitro and in a mouse model assessing subcutaneous tumor growth of A549 cells transfected into nude mice. Importantly, the proliferation-enhancing effect of LPS was also evident in ex vivo stimulated intact human tissue specimen obtained from patients with NSCLC, thus giving a strong hint on the clinical significance of the current data.

Proliferation of tumor cells in response to LPS in A549 cells was induced by the ligation of LPS to CD14 and the pattern recognition receptor TLR4 followed by COX-2-dependent PGE_2_ synthesis. Moreover, activation of EGFR was involved in LPS-activated tumor cell proliferation.

Our data suggest that LPS is a relevant and potent stimulator of tumor cell proliferation. In vitro, a clear dose- and time-dependent increase in MTS activity was noted in A549 cells incubated with various concentrations of LPS. The increase in metabolic activity was already noted after 24 h of LPS treatment and reached a maximum after 48 h. The metabolic activity of A549 cells is known to correspond to cellular proliferation [31], which was further validated by automatic cell counting with cell numbers being doubled after 24 h of LPS stimulation and further climbing thereafter, but to a lesser extent than MTS activity. As the best correlation between MTS activity and cell counts was evident after 24 h of stimulation, this time point was chosen for further experiments. In A549 cells, both the widely used LPS from *E. coli* 0111:B4 strain and a highly purified LPS preparation from *E. coli* strain F515 were equally effective, supporting the notion that this effect is not restricted to LPS of a certain bacterial strain or due to any contamination or preparation effect. Moreover, this proliferation- enhancing effect of LPS was not restricted to A549 cells. Intact human NSCLC tissue specimens that were stimulated ex vivo using the STST assay exhibited an increase in Ki-67 expression, a marker of cellular proliferation. The currently used HOPE-fixation technique for NSCLC specimen provides an excellent preservation of proteins and extremely low degradation of nucleic acids [29], thus allowing to illustrate most solidly the intact tumor’s response to endotoxin. Additionally, in vivo tumor size was doubled in LPS-challenged tumors in the A549 adenocarcinoma mouse model. These results are in accordance with recent experimental data showing that LPS stimulates tumor growth in human ovarian cancer [42] and animal models of breast cancer [43] and induces apoptosis resistance of NSCLC cells [44].

The potential biologic significance of the proliferation enhancing effects of LPS are supported by the fact that the extent of LPS-induced cellular proliferation observed in our study approached or even exceeded that reported by well known other endo- or exogenous proliferative agents such as IL-8 [45] or benzo[a]pyrene [46]. It is noteworthy that even the single administration of low doses of LPS to A549 cells before subcutaneous implantation in the A549 adenocarcinoma mouse model was sufficient to induce a doubling of tumor size within the 12-day observation period.

As the currently described short-time tumor-promoting effects of endotoxin detected in vitro and in the subcutaneous tumor model might not reflect the physiological situation exactly, the effects of endotoxin and the potential mechanisms (i.e., TLR4, EGFR, COX-2 activation,) need to be validated in orthotopic lung cancer models, for example, in recently described in situ models using bioluminescence [47, 48].

However, our current experimental data clearly suggest that binding of LPS to the LPS receptors CD14 and TLR4 appears to be the initial steps in LPS-induced increase in proliferation. Application of both the blocking anti-CD14 antibody and the anti-TLR4 antibody suppressed the LPS-induced increase in MTS activity in A549-cells, whereas addition of the anti-TLR2 antibody was ineffective. This is of particular interest, as an up-regulation of TLR4 expression has been reported recently in human adenocarcinoma of the lung with TLR4 expression levels correlating with malignancy [26].

In our study, binding of LPS to CD14 and TLR4 was followed by the activation of COX-2 with subsequent PGE_2_ release. CD14/TLR4-dependent COX-2 activation may represent a crucial step in mediating tumor proliferation in response to endotoxin, and this conclusion is based on several reasons: first, when CD14 or TLR4 (but not TLR2) were blocked by the respective antibodies, no PGE_2_ was released from A549 cells and MTS activity as a marker of cell proliferation remained unchanged; second, when the non-specific COX inhibitor indomethacin was used, neither PGE_2_ release nor an increase in MTS activity was noted in LPS-stimulated A549 cells; third, the COX-2-specific inhibitor NS398 was equally effective in inhibiting prostanoid formation and MTS activity as compared to indomethacin; and fourth, marked expression of COX-2 mRNA in both A549 cells and in intact human ex vivo stimulated human lung cancer tissue was observed, following incubation with LPS. Therefore, COX-2 is strongly suggested as the predominant isoform of COX engaged in mediating tumor cell proliferation. Whether COX-2-derived PGE_2_ is the key prostanoid mediating LPS-induced NSCLC proliferation cannot directly be derived from our data. However, PGE_2_ is the major prostanoid of A549 cells [16], is strongly induced in lung cancer tissue [15] and is known to promote NSCLC growth in vitro [11] and in vivo in a murine model of lung cancer [38].

In addition to CD14-and TLR4-dependent LPS-signaling, activation of the EGFR seems to be a crucial element of the observed tumor cell proliferation. When EGFR was blocked by cetuximab in A549 cells, both the LPS-induced increase in MTS activity and the release of PGE_2_ were blunted. This is of special interest, since overexpression of EGFR has been associated with tumor development and poor prognosis in NSCLC [49, 50] and novel inhibitors of this signaling pathway such as cetuximab, erlotinib and gefitinib are of increasing clinical importance [51–54]. Thus, EGFR inhibitors may equally be effective in inhibiting NSCLC growth triggered by infections.

The precise mechanism of LPS-induced EGFR activation cannot be derived from our data. Direct activation of this receptor system and downstream signaling events such as ERK and JNK activation in response to LPS have been described [55, 56]. In addition, we and others have demonstrated that LPS activates IL-8 synthesis in A549 cells [57, 58], and this cytokine is known to transactivate EGFR in NSCLC cell lines as an alternative pathway [59].

In conclusion, this is the first study to demonstrate that LPS effectively induces tumor growth in various experimental models of NSCLC in vitro, ex vivo and in vivo via CD14-, TLR4-, EGFR- and COX-2-signaling. Our results support the hypothesis that pulmonary infections may severely worsen the prognosis of NSCLC by accelerating tumor progression.
